# Superstitions about Cats

**Published:** 1881-02

**Authors:** 


### SUPERSTITIONS ABOUT CATS.

The cat has been connected with many curious superstitions in various parts of the world. In some localities, for instance, it is believed that witches in the shape of cats are in the habit of roaming about the roofs of the houses during the month of February ; hence they were promptly shot. In Germany, also, a similar notion prevails respecting black cats, in consequence of which they are never allowed to go near the cradles of young children, though it is not easy to understand why the young should be more exposed to danger from these suppositious witches than those more advanced in years. Numerous instances might be given of the incredible nonsense that has been believed and is believed about cats. In Sweden the peasants have a deep-rooted faith in the cat carrier. This belief is traceable to the seventeenth century. At this period there were seventy tried who made nearly the following declaration : After compelling them to sign a compact with him, the devil often served hearty dinners, followed by games and wrestling. When he was in good humor he thrashed them with a long pole, after which he laughed heartily, or played the harp, in order to divert them and make them dance. They added that he made each one of them a present of a cat endowed with certain gifts. This cat comprehended all that was said to it, and would go out and steal objects that were indicated, without the possessor being able to perceive him. At the present time the peasant does not complain whatever object is stolen from him. They attribute the theft to a cat carrier. In Sicily, where the cat is looked upon as sacred to St. Martha, there is a superstition that any one who willfully or accidentally kills a cat will be punished by the serious retribution of seven years' unhappiness. Melton, in his "Astrologaster," says: "When the cat washes her face over her eares, we shall have great store of raine." The sneezing of a cat was considered a lucky omen to a bride who was to be married the next day. In Wilsford's "Nature Secrets," "cats coveting the fire more than ordinary, or licking their feet and trimming hair of their heads and mustachios, presages rainy weather." In the "Statistical Accounts of Scotland," it is stated that if a cat was permitted to leap over a corpse, it portended misfortune. Their tenacity of life comes out in the proverb, "Care killed the cat." Their familiar presence at every one's hearth is alluded to in "A cat may look at a king."

